# Effect of universal test and treat on retention and mortality among people living with HIV-infection in Uganda: An interrupted time series analysis

**DOI:** 10.1371/journal.pone.0268226

**Published:** 2022-05-17

**Authors:** Levicatus Mugenyi, Mastula Nanfuka, Jaffer Byawaka, Collins Agaba, Andrew Mijumbi, David Kagimu, Kenneth Mugisha, Jaffer Shabbar, Michael Etukoit

**Affiliations:** 1 The AIDS Support Organization, Kampala, Uganda; 2 Medical Research Council/Uganda Virus Research Institute & London School of Hygiene and Tropical Medicine, Entebbe Unit, Entebbe, Uganda; 3 Liverpool School of Tropical Medicine, Liverpool, United Kingdom; University of the Witwatersrand, SOUTH AFRICA

## Abstract

**Background:**

Few studies have analysed the effect of HIV universal test and treat (UTT) on retention and mortality among people living with HIV (PLHIV) in routine care. We examined six-month retention and mortality at 11 health facilities (HFs) run by a large NGO, The AIDS Support Organisation (TASO), before and after UTT.

**Methods:**

We used a quasi-experimental study using patient data extracted from 11 TASO HFs. Two periods, one before UTT (2015–2016) and the other during UTT (2017–2018) were compared. The primary outcome was six-month retention defined as the proportion of PLHIV who were alive and in care at six months from enrolment. The secondary outcome was six-month mortality defined as the proportion of PLHIV who died within six months from enrolment. We performed an interrupted time series analysis using graphical aids to study trends in six-month retention and mortality and a segmented regression to evaluate the effect of UTT. We used a generalized linear mixed model (GLMM) and generalized estimating equations (GEE) to account for facility-level clustering.

**Results:**

Of the 20,171 PLHIV registered between 2015 and 2018 and included in the analysis, 12,757 (63.2%) were enrolled during the UTT period. 5256/7414 (70.9%) of the pre-UTT period compared to 12239/12757 (95.9%) of the UTT were initiated on ART treatment with 6 months from enrolment. The median time from enrolment to initiating ART was 14 (interquartile range (IQR): 0–31) days for the pre-UTT compared to 0 (IQR: 0–0) days for the UTT period. The median age at enrolment was 32.5 years for the pre-UTT and 35.0 years for the UTT period. Overall, the six-month retention just after scale-up of UTT, increased by 9.2 percentage points (p = 0.002) from the baseline value of 82.6% (95% CI: 77.6%-87.5%) but it eventually decreased at a rate 1.0 percentage point (p = 0.014) for cohorts recruited each month after UTT. The baseline six-month mortality was 3.3% (95% CI: 2.4%-4.2%) and this decreased by 1.6 percentage points (p = 0.003) immediately after UTT. The six-month mortality continued decreasing at a rate of 0.1 percentage points (p = 0.002) for cohorts enrolled each month after UTT. Retention differed between some health facilities with Rukungiri HF having the highest and Soroti the lowest retention. Retention was slightly higher among males and younger people. Mortality was highest among people aged 50 years and more. The effect of UTT on retention and mortality was similar across sex and age groups.

**Conclusion:**

Overall, UTT significantly led to an immediate increase in retention and decrease in mortality among PLHIV enrolled in HIV care from 11 HFs run by TASO in Uganda. However, retention (and mortality) significantly decreased for cohorts enrolled each month after UTT. Retention was highest in Rukungiri and lowest in Soroti HFs and slightly higher in males and younger people. Mortality was highest in older patients and lowest in adolescents. We recommend for innovative interventions to improve the overall retention particularly in facilities reporting low retention in order to achieve the UNAIDS 2030 target of 95-95-95.

## Introduction

Screening populations at risk for HIV-infection and offering rapid treatment with antiretroviral therapy (ART) in those who are diagnosed as HIV positive (“universal test-and-treat” (UTT)) is standard practice and a key strategy of the World Health Organization (WHO), geared towards ending the HIV epidemic [1]. We refer to UTT as screening all population at risk and rapidly initiating on ART all those diagnosed HIV positive regardless of their CD4 count and WHO clinical stage.

UTT has been scaled up actively in high HIV-burden countries since around 2014–2015 [1, 2]. A study that evaluated UTT in four randomized population-based trials conducted in Botswana, Zambia, South Africa, Kenya and Uganda demonstrated that UTT increased viral suppression in all trials [3–6], decreased HIV incidence in two trials [5, 6], and decreased mortality in one trial [3] that had released information on HIV-associated mortality [7]. For example, in the Botswana and South Africa/Zambia trials, the annual HIV incidence was approximately 20% to 30% lower in the intervention (which included universal testing) compared to control arms (no universal testing) [5–7]. On the other hand, in the SEARCH trial conducted in Kenya and Uganda (where both arms had universal testing), HIV incidence declined by 32% over three years [7]. In addition, UTT reduced HIV associated mortality by 23% in the intervention versus control communities in the SEARCH trial [7], a different study conducted in KwaZulu-Natal in South Africa showed that UTT did not lead to reduction in HIV incidence and this was attributed to poor linkage to care [4].

In Uganda, it is estimated that 84% of HIV-infected persons know their status, and of these 72% are on antiretroviral therapy and of these 64% are virally suppressed [8]. There has been 58% decrease in deaths attributed to HIV-infection from 58,000 in 2010 to 23,000 in 2018 [8]. In 2016, Uganda expanded the test and treat policy from the 2013 guidelines and this policy was rolled out across the country in 2017 [9].

For countries in Africa to achieve the epidemic control, retention in care of those tested and started on antiretroviral therapy is critical. Factors documented to facilitate retention in care include older age, decreased time between testing and ART initiation, higher CD4 count at ART initiation (among men), maintaining physical health, a patient-centered treatment environment, supportive partnerships, few negative consequences to disclosure, and the ability to seek care in facilities outside of their community of residence [10]. Lack of psychological support [11] and being asymptomatic with high CD4 count [7] are some of the documented barriers to ART retention. In our study setting, it is claimed that with UTT strategy, psychological support could have reduced substantially since patients receive treatment almost immediately following diagnosis (unpublished). Could the UTT strategy negatively affect retention? A study conducted in Malawi reported the reverse, with 12 months retention of 83.0% in the UTT cohort compared to 76.2% in the pre-UTT cohort [12]. Similarly, another study conducted in Swaziland that compared retention between patients in the pre-UTT and UTT cohorts at 12 months reported retention rates at 80% and 86%, respectively [13]. Similar findings from a test and treat trial (SEARCH) conducted in Uganda and Kenya also show that a decreased time between testing and ART initiation was associated with higher rates of retention [10].

Despite the above information on improvement in retention following UTT, the strategies to increase HIV yield and starting patients on treatment while in communities [14] without proper counselling and linking into care in facilities preferred by the patients can increase loss to follow-up (LTFU) and transfer-outs, which could negatively affect retention. This implies that though UTT decreases mortality [7], there is a likelihood of increased LTFU and transfer-outs especially during the scale-up period which may negatively affect retention. Although some studies have reported higher retention during UTT, they either followed smaller cohorts and based their findings on one or fewer health facilities [10, 12] or their findings were based on well monitored trials [7, 10] other than program data generated from a single organization managing a big number PLHIV on ART at 11 health facilities yet with limited resources. The question is, could UTT have negatively affected retention which the ART programmes had achieved at The AIDS Support Organization (TASO) prior to UTT?.

In this paper, we aimed to determine the effect of UTT strategy on retention and mortality among PLHIV receiving care from 11 health facilities (HFs) in Uganda. We hypothesize that UTT could have negatively affected retention which the ART programmes had achieved in TASO HFs prior to UTT policy.

## Materials and methods

### Study design and setting

We used a quasi-experimental study to compare two periods, that is, before and after UTT. We extracted and used secondary data for PLHIV registered between 2015 and 2018 at TASO-Uganda using a pre-existing cohort electronic database. TASO is one of the pioneer nongovernmental organizations in HIV service delivery in Uganda that has provided HIV services, health systems strengthening, community and economic strengthening activities for PLHIV since its inception in 1987 (34 years). It is located within the Mulago National Referral Hospital complex, approximately 5 kilometers north-east of Kampala’s central business district. TASO serves over 36 districts of Uganda through its 11 regional service delivery centers (health facilities) funded mainly by PEPFAR and CDC. TASO has provided HIV prevention, care and treatment services to over 180,000 PLHIV since its inception. In addition to trained health workers (doctors, nurses and councilors), TASO practices meaningful involvement of PLHIV in their own chronic care to ensure ownership. In this case, HIV patients, known as expert clients or peer educators, are actively involved in HIV service delivery through a peer-to-peer approach by providing peer-to-peer support to fellow PLHIV. For one to qualify to be an expert client or peer educator, he/she should be exemplary by having high adherence and be virally suppressed. The newly initiated patients attend clinics and pick their ART drugs nearly on a monthly basis and subsequently every after 3 to 6 months for the virally suppressed patients. TASO rolled out UTT across all its 11 health facilities in 2017 at the same time as the national policy.

We evaluated two periods, one pre-UTT (2015–2016) and the other post-UTT (2017–2018). During the pre-UTT period, HIV positive patients were being initiated on ART when their CD4 count was less than 500 *cells*/μL with exception of pregnant women, children below 15 years and key populations who were started on ART regardless of their CD4 count [15].

### Study participants

We included all PLHIV enrolled in care between 2015 and 2018. We excluded children below 15 years, pregnant mothers and key populations sub-groups to avoid bias in comparing the two periods since these were initiated on ART regardless of their CD4 count by TASO before and after UTT [15]. Key populations included sex workers, trucker drivers, fisher-folk, incarcerated, men who have sex with men (MSM), uniformed person, migrant worker, internally displaced persons, people who inject drugs, transgender, client of sex worker, and discordant couples. We also excluded in the analysis all patients who transferred care out of TASO because we could not tell if these were retained in care or were lost to follow-up.

### Outcomes

The primary outcome of interest was six-month retention defined as proportion of PLHIV who were alive and in care at six months from enrolment. In this study, enrolment in care refers to getting registered in TASO as HIV patient after or before initiating ART treatment. The secondary outcome was six-month mortality defined as proportion of PLHIV who died from all causes not necessarily linked to HIV within six months from enrolment. Patients not seen more than 3 months from their next appointment date were considered as LTFU. Both retention and mortality were binary outcomes.

### Statistical analysis

We generated a consort chart showing the number of PLHIV enrolled at the 11 TASO HFs between 2015 and 2018, number excluded and number experiencing study outcomes stratified by the two study periods. Six-month retention was calculated considering different cohorts of PLHIV enrolled each month from January 2015 through December 2018 resulting in six-month retention trend data. This implies that the first time series data point refers to six-month retention for the cohort enrolled in January 2015, the second six-month retention data point refers to the cohort enrolled in Feb 2015 and so on. For each month cohort, the denominator for retention equals to the number of PLHIV enrolled in that month (after exclusions) and followed for six months, and the numerator equals to the number that remained in care at the end of 6 months from enrolment. We also analysed for trend in six-month mortality. In this case, the numerator equals to the number known to have died within 6 months from enrolment and the denominator is as described for retention. We used interrupted time series analysis to evaluate the effect of UTT on retention and mortality using the six-month rates.

Line graphs were used to show trends in six-month retention and six-month mortality comparing the two periods. We used segmented regression analysis to evaluate the effect of UTT on retention and mortality. The segmented regression model was fitted as follows [16];

$$Y_{t}=\beta_{0}+\beta_{1}*{Time}_{t}+\beta_{2}*{Period}_{t}+\beta_{3}*{Time\;after\;UTT}_{t}+e_{t},$$

where *Y*_*t*_ is six-month retention (or mortality); *Time* is a continuous variable for time in months starting from January 2015; *Period* is an indicator for the study period (pre-UTT = 0, UTT = 1); *Time after UTT* is a continuous variable counting the number of months from when UTT was fully scaled-up (January 2017 which is month 25 in the series) with values starting from 1 for the UTT period and with value 0 everywhere during the pre-UTT period; *β*_0_ estimates the baseline retention (or mortality) (intercept): baseline refers to the first month time point, which is January 2015; *β*_1_ estimates change in retention (or mortality) that occurs for each month cohort before UTT; *β*_2_ estimates the level change just after UTT; *β*_3_ estimates the change in retention (or mortality) after introduction of UTT; and *e*_*t*_ is an error term representing the random variability not explained by the model. We used a generalized linear mixed model (GLMM) to account for facility-level clustering and to be able to predict facility-specific retention rates. For mortality, we used generalized estimating equations (GEE) because the GLMM failed to converge possibly due to very few or no death in some facilities for patients enrolled in some months. We fitted both the GLMM and the GEE models to binary data representing retention and death considering a Gaussian family distribution, an identity link function and robust standard errors. The choice for the Gaussian family distribution with robust standard errors (over the commonly used logistic regression), enabled us to directly estimate the retention and mortality rates (proportions) and difference in rates [17], noting that the mean of binary data is a rate (proportion). The models were fitted overall and separately for males, females and age groups. In the latter case (sub-analysis), we first obtained the six-month retention and mortality data separately for males, females and for the different age groups and later we fitted segmented models to these data for each category. All variables included in the models had complete data, except age in the UTT period with only 0.05% missing data, which was a very small percentage to alter results. Therefore, complete case analysis was considered. All analyses were done using STATA version 14 software [18] and R software [19].

### Ethics

The study received a waiver for the requirement to obtain informed consent from the TASO Institutional Review Board because it used fully anonymized and already existing data (TASOREC/096/19-UG-REC-009).

## Results

There were 26,255 PLHIV enrolled into 11 TASO health facilities between 2015 and 2018 of whom 16,244 (61.9%) were enrolled during the UTT period. After applying the exclusion criteria, 20,171 PLHIV were included in the analysis with 12,757 (63.2%) enrolled during the UTT period. Of those enrolled during the pre-UTT period and included in the analysis, 5256/7414 (70.9%) were started on ART within 6 months from enrolment with a median time of 14 (interquartile range (IQR): 0–31) days compared to 12239/12757 (95.9%) started on ART within a median of 0 (IQR: 0–0) days for those enrolled during the UTT period. The median duration on ART in 6 months from enrolment was 5.5 (IQR: 4.9–6.0) months for those enrolled during pre-UTT period and 6 (IQR: 6–6) months for the UTT period.

In Table 1, we show the baseline characteristics of the PLHIV enrolled in care during the two periods that were included in the analysis. More than half (60.6%) were females and this was similar across the two periods. The median age (IQR) at enrolment was 32.7 (26.8–40.5) years for the pre-UTT cohort and 35.1 (28.1–43.9) years for the UTT cohort (p<0.001).

**Table 1:** Baseline characteristics of PLHIV enrolled during the two study periods, pre and during UTT.

	Total	Pre-UTT (2015–2016) n (%)	UTT (2017–2018) n (%)
	N = 20,171	N = 7,414	N = 12,757
**Sex:** Female	12,226 (60.6)	4,573 (61.7)	7,653 (60.0)
Age group (years)*:			
Median (IQR)	34.2 (27.6–42.7)	32.7 (26.8–40.5)	35.1 (28.1–43.9)
15–17 (adolescents)	304 (1.5)	123 (1.7)	181 (1.4)
18–24 (young people)	2,997 (14.9)	1,253 (16.9)	1,744 (13.7)
25–49 (middle-age)	14,521 (72.0)	5,327 (71.9)	9,194 (72.1)
50+ (old people)	2,343 (11.6)	711 (9.6)	1,632 (12.8)
Missing	6 (0.03)	0 (0.0)	6 (0.05)
**TASO health facilities:**			
Entebbe	1,827 (9.1)	973 (13.1)	854 (6.7)
Gulu	2,371 (11.8)	793 (10.7)	1,578 (12.4)
Jinja	1,870 (9.3)	459 (6.2)	1,411 (11.1)
Masaka	2,128 (10.6)	947 (12.8)	1,181 (9.3)
Masindi	1,159 (5.8)	434 (5.9)	725 (5.7)
Mbale	2,570 (12.7)	817 (11.0)	1,753 (13.7)
Mbarara	1,902 (9.4)	824 (11.1)	1,078 (8.5)
Mulago	2,021 (10.0)	891 (12.0)	1,130 (8.9)
Rukungiri	1,222 (6.1)	542 (7.3)	680 (5.3)
Soroti	860 (4.3)	263 (3.6)	597 (4.7)
Tororo	2,241 (11.1)	471 (6.4)	1,770 (13.9)
**Education:**			
None/pre-primary	2,773 (13.7)	893 (12.0)	1,880 (14.7)
Primary	11,093 (55.0)	3,886 (52.4)	7,207 (56.5)
Secondary	4,889 (24.2)	2,034 (27.4)	2,855 (22.4)
Higher Institution	1,191 (5.9)	541 (7.3)	650 (5.1)
Other	67 (0.3)	25 (0.3)	42 (0.3)
Missing	158 (0.8)	35 (0.5)	123 (1.0)
**Occupation:**			
None	2,024 (10.0)	828 (11.2)	1,196 (9.4)
Paid employee	1,622 (8.0)	650 (8.8)	972 (7.6)
Peasant	6,853 (34.0)	2,004 (27.0)	4,849 (38.0)
Casual laborer	3,519 (17.4)	1,359 (18.3)	2,160 (16.9)
Housewife	1,168 (5.8)	376 (5.1)	792 (6.2)
Vendor/business person	3,808 (18.9)	1,709 (23.1)	2,099 (16.5)
Dependent	98 (0.5)	23 (0.3)	75 (0.6)
Student	57 (0.3)	30 (0.4)	27 (0.2)
Other	895 (4.4)	407 (5.5)	488 (3.8)
Missing	127 (0.6)	28 (0.4)	99 (0.8)
**Marital status:**			
Married monogamous	8,654 (42.9)	3,035 (40.9)	5,619 (44.0)
Married polygamous	1,076 (5.3)	337 (4.5)	739 (5.8)
Cohabiting	1,258 (6.2)	431 (5.8)	827 (6.5)
Separated	4,757 (23.6)	1,918 (25.9)	2,839 (22.3)
Divorced	380 (1.9)	118 (1.6)	262 (2.1)
Widowed	1,748 (8.7)	606 (8.2)	1,142 (9.0)
Never married	2,028 (10.1)	892 (12.0)	1,136 (8.9)
Emancipated minor	115 (0.6)	49 (0.7)	66 (0.5)
Other	26 (0.1)	9 (0.1)	17 (0.1)
Missing	129 (0.6)	19 (0.3)	110 (0.9)

*6 missing age and all in the UTT period.

**Fig 1:**
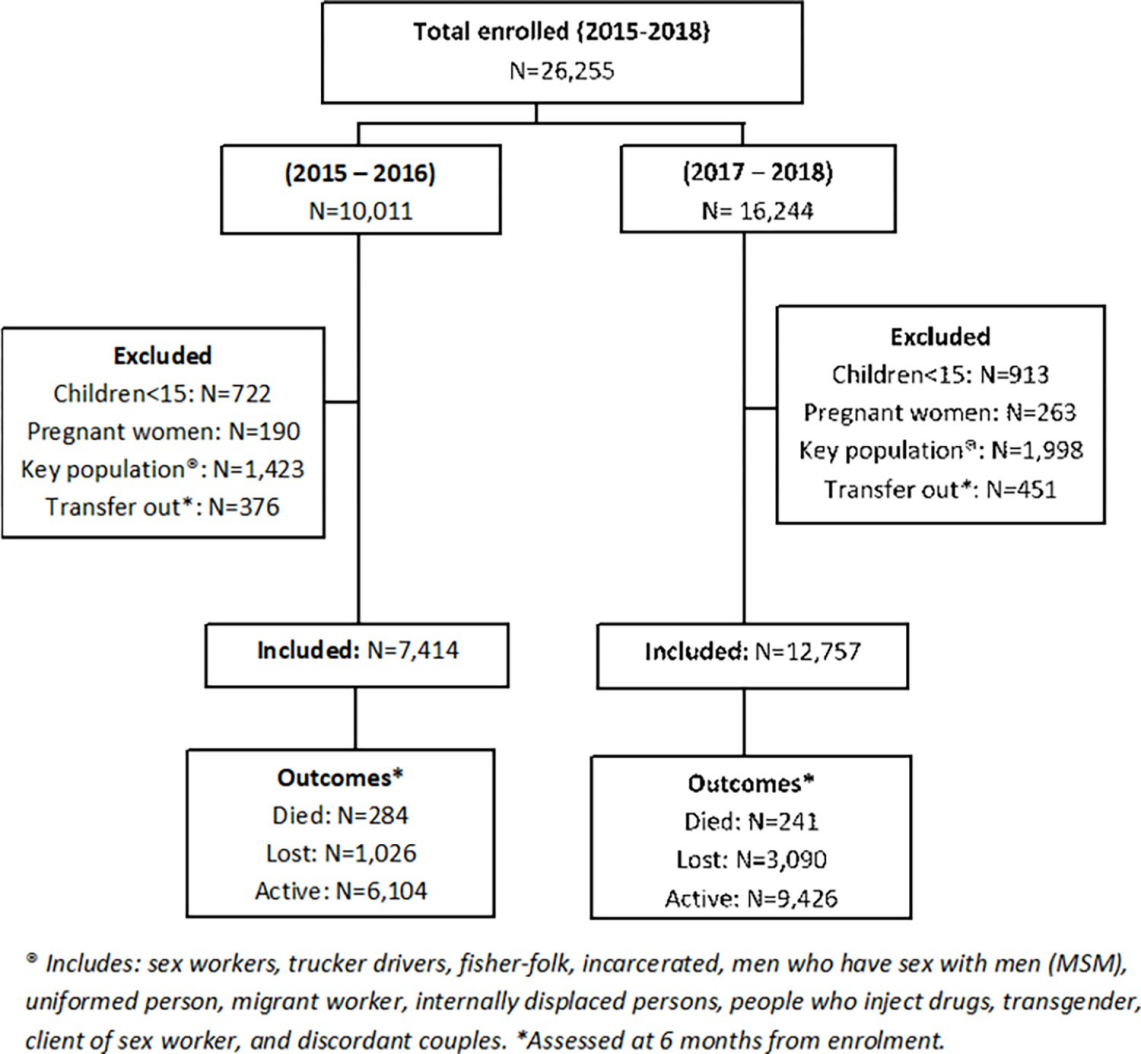
Consort chart showing number of HIV patients and their six months follow up outcomes at 11 TASO health facilities in Uganda for PLHIV enrolled in care in the period 2015–2018.

Fig 1 shows a consort chart for the number of PLHIV enrolled at 11 TASO health facilities between 2015 and 2018 and the study outcomes at 6 months from enrolment stratified by the two periods. Of those enrolled during the pre-UTT period and were included in the analysis, 3.8% died, 13.8% got lost to follow-up, and 82.3% were alive and active in care at 6 months. Similarly, of those enrolled and included in the UTT period, 1.9% died, 24.2% got lost, and 73.9% were alive and active in care at 6 months. Note that in Fig 1, there is an overlap in numbers in the excluded box. This means that number enrolled minus the total in the excluded box does not necessarily equal to the number in the included box.

**Fig 2:**
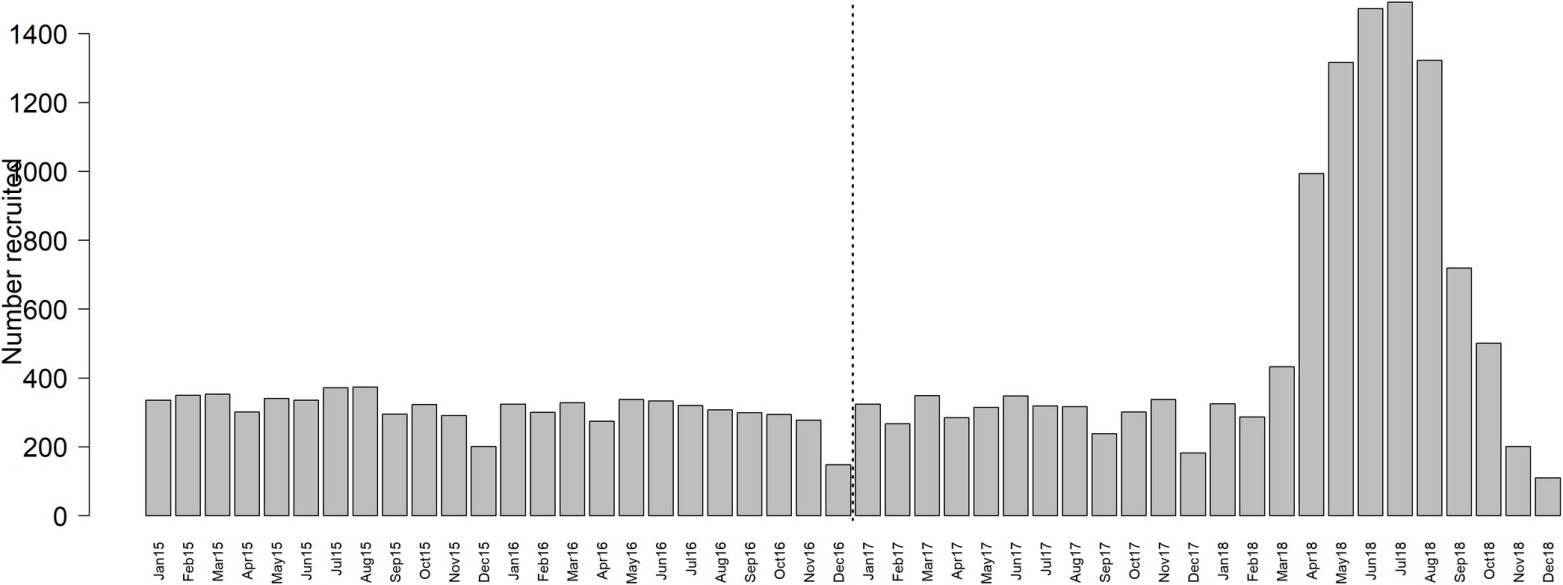
Bar graph showing number of HIV patients enrolled in each month from January 2015 through December 2018 comparing pre-UTT (2015–2016) and UTT (2017–2018) periods separated by a vertical dotted line.

Fig 2 shows a bar graph for the number of PLHIV enrolled in each month from January 2015 through December 2018 and were included in the analysis for retention and mortality. The number enrolled each month between January 2015 and February 2018 fluctuated around 300 patients except for the month of December where it was about 200. The number enrolled increased steadily (during UTT period) from 433 in March 2018 to a peak of 1,473 in July 2018 after which it declined to 110 in December 2018.

**Fig 3:**
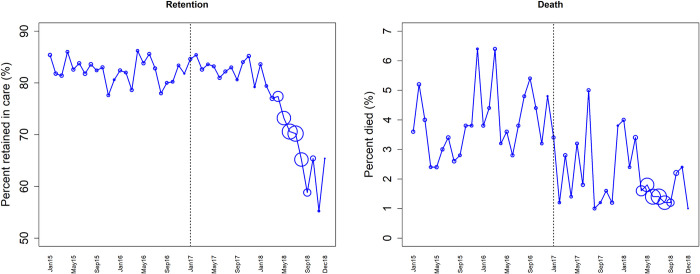
Time series trends showing six-month retention and six-month mortality for cohorts enrolled in each month from January 2015 through December 2018 comparing pre-UTT (2015–2016) and UTT (2017–2018) periods separated by a vertical dotted line. The size of the circles corresponds to the number of HIV patients enrolled and included in the analysis.

Fig 3 shows the time series for observed retention and mortality rates calculated at six months from enrolment for cohorts enrolled each month from January 2015 through December 2018 and were included in the analysis. The dotted vertical line indicates a point (January 2017) when test and treat was fully scaled up in all 11 TASO health facilities. From the figure, retention fluctuates around 82% during the pre-UTT period and in the first year of UTT after which it declines steadily to 55% for the cohort recruited in November 2018. The six-month mortality was lower during UTT oscillating between 0.9% and 5.0% compared to 2.3% and 6.5% before UTT. The figure shows a marked reduction in retention over time after UTT is introduced with little to moderate reductions in mortality.

Table 2 shows results from the segmented regression models fitted to six-month retention and mortality data. The results indicate that just before UTT, the six-month retention was 82.6% (95% confidence interval (CI): 77.6%-87.5%) and there was no significant month-to-month change in retention before UTT (p = 0.527). Just after the scale up of UTT, retention increased by 9.2 percentage points (p = 0.002). There was a significant decrease in the month-to-month trend in retention by 1.0 percentage point after UTT (p = 0.014). There was a small variability between facilities (variance = 0.01). The baseline six-month mortality before UTT was 3.3% (95% CI: 2.4%-4.2%) and this increased by 0.1 percentage points, though not significant (p = 0.091). Just after UTT, mortality significantly decreased by 1.6 percentage points (p = 0.003). The month-to-month trend in mortality decreased by 0.1 percentage points (p = 0.002).

**Table 2:** Parameter estimates, standard errors and p-values from the segmented regression models predicting six-month retention and mortality among PLHIV enrolled in the 11 TASO health facilities between 2015 and 2018.

Effect	Coefficient	Standard error	z-statistic	p-value
Retention (GLMM)				
Intercept *β*_0_	0.826	0.025	32.65	<0.001
Baseline trend *β*_1_	-0.001	0.001	-0.63	0.527
Level change after UTT *β*_2_	0.092	0.030	3.06	0.002
Trend change after UTT *β*_3_	-0.010	0.004	-2.46	0.014
Variance (facility)	0.010	0.005		
Mortality (GEE)				
Intercept *β*_0_	0.033	0.006	7.49	<0.001
Baseline trend *β*_1_	0.001	0.0004	1.69	0.091
Level change after UTT *β*_2_	-0.016	0.006	-2.93	0.003
Trend change after UTT *β*_3_	-0.001	0.0004	-3.06	0.002

**Fig 4:**
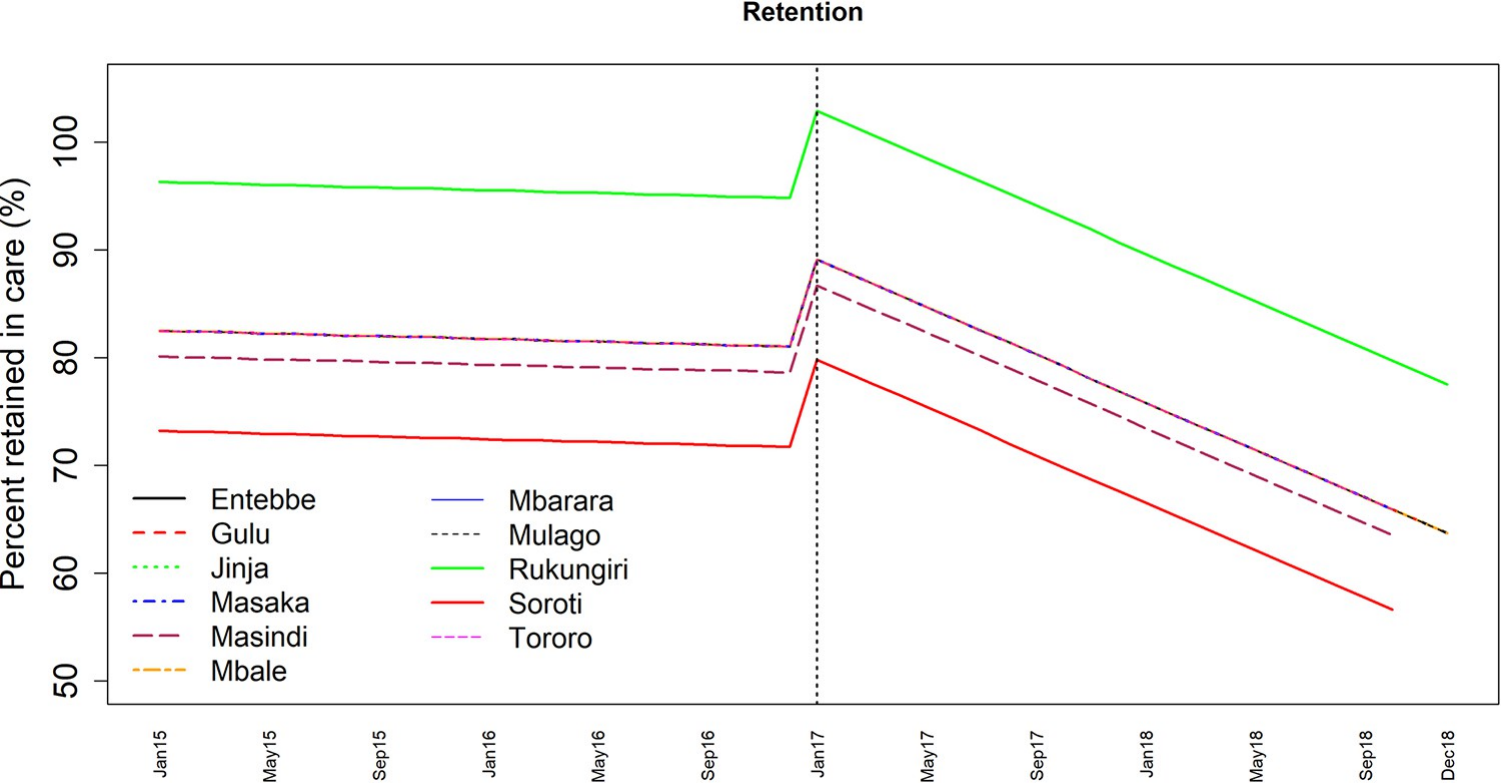
Time series trends showing predicted six-month retention for cohorts enrolled in each month from January 2015 through December 2018 comparing pre-UTT (2015–2016) and UTT (2017–2018) periods separated by a vertical dotted line.

Fig 4 shows facility-specific predicted six-month retention rates obtained after fitting the GLMM. Retention before and after UTT was highest in Rukungiri and lowest in Soroti health facilities. Masindi health facility was next to Soroti with lower rates of retention. There was no difference in retention between Entebbe, Gulu, Jinja, Masaka, Mbale, Mbarara, Mulago and Tororo health facilities.

When analysed separately for males and females accounting for facility differences, the baseline retention was 79.8% (95% CI: 71.0%-88.6%) among females and 81.0% (95% CI: 75.0%-87.0%) among males. When analysed separately for age groups accounting for facility differences, the baseline retention was 89.0% (95% CI: 80.8%-97.3%) for adolescents (15–17 years); 81.9% (95% CI: 75.4%-88.3%) for 15–24 years; 80.7% (95% CI: 75.7%-85.8%) for 25–49 years; and 81.4% (95% CI: 73.7%-89.2%) for 50 years and more. The increase in retention just after the scale-up of UTT as well as the rates of decrease in retention after UTT were similar cross sex and age groups and were equal to the overall rates shown in Table 2. The baseline mortality was higher in males compared to females (3.8% (95% CI: 2.2%-5.4%) vs 3.0% (95% CI: 1.8%-4.2%)). Also, the baseline mortality was highest among those aged 50 years and more (5.0% (95%CI: 2.0%-7.9%)) followed by those aged 25–49 years (3.5% (95%CI: 2.2%-4.8%)) and lowest among the younger people aged 15–24 years (1.6% (95%CI: 0.4%-2.8%)). Similarly, rates of decrease in mortality following UTT were comparable across sex and age groups and were equal to the overall rates presented in Table 2.

## Discussion

Our study has shown that UTT for PLHIV in routine care in Uganda had a significant effect on both retention and mortality. Overall, there was no significant month-to-month change in retention and mortality before UTT. The six-month retention and six-month mortality were respectively rather stationary at 82.6% and 3.3% for the period before UTT. However, after scale up of UTT, there was a sudden increase in retention and later a decrease in month-to-month trend in retention. Mortality suddenly decreased after UTT and the trend continued decreasing each month. The effect of UTT on both retention and mortality was similar across sex and age groups.

The sudden increase in retention of 9.2 percentage points just after UTT agrees with the study conducted in Malawi which reported a 6% higher 12-months retention among UTT patients compared to the standard of care [12]. Also, our findings on the sudden increase in retention are not different from those by Brown *et al*. who showed in a study conducted in Uganda that delays in starting on ART treatment was associated with non-retention [10, 20].

The decrease in retention each month after full scale-up of UTT in our study setting could be attributed to the increased number of patients started on ART and enrolled in care which could have burdened the available resources. In addition, during UTT many patients were being enrolled in TASO from communities [14] without proper counselling and linking into care in facilities preferred by the patients, this could have increased LTFU hence decreasing retention. Also, our study emphasizes the findings from South Africa by Arnesen *et al*. who reported higher ART discontinuation rates and hence poor retention among males and patients aged 18–35 years [21].

Our findings further indicated that the effect of UTT on retention differed between health facilities where patients get HIV care. This could be due to increased number of PLHIV enrolled in care during UTT with limited resources in some facilities which could have affected service delivery and consequently the retention. Moreover, the campaign to increase HIV yield during the period of UTT by testing people from communities and start those who test positive on treatment without proper referral and close monitoring could also have increased LTFU and hence affect retention.

Overall, our study shows higher rates of retention for PLHIV compared to other studies conducted elsewhere [22–24]. For the past 34 years, TASO, using its experienced and dedicated health workers, counsellors and peer educators has been closely monitoring, and providing intensive counselling and continuous education to PLHIV under TASO care, a unique support different from elsewhere. This could explain the higher retention reported in this paper compared to other studies conducted in Ethiopia [22], Mozambique [23], and Cameroon [24]. However, the retention rates reported in our study are lower than those reported in the SEARCH study conducted in Kenya and Uganda [20] and this could be attributed to the large number of patients enrolled in HIV care each month visa-vie the available resources. Also, the large campaign of testing and bringing into care all those that tested positive during that period could explain the low retention rates since people were probably not prepared enough to initiate treatment. This could also explain the decline in retention during UTT that is reported in this paper.

The baseline mortality estimated in this study is comparable to that from a similar study conducted in Ethiopia which estimated an incidence of death of 3 per-100 person years [25]. In addition, both studies show a significant decline in mortality following UTT [25]. Our finding on reduction in mortality after UTT agrees with the results from the SEARCH study which was conducted in Uganda and Kenya that reported a 23% reduction in HIV associated mortality following UTT.

The reduction in mortality following UTT could be attributed to the benefits of starting treatment in early stages of the infection [1]. Early treatment has been documented to prevent the development of fatal opportunistic infections and it increases survival of patients [26]. The higher mortality rate among males compared to females may be attributed to poor medical seeking practices and late ART initiation among men compared to women [27].

The strength of our study over that of Malawi and that by Brown *et al* in Uganda is that we used interrupted time series analysis using six-month rates generated from cohorts of PLHIV enrolled each month and followed for at least six months other than simply comparing the pre-UTT and UTT as ordinary cohorts. In addition, we used data from multi-sites enabling us to account for site differences using GLMM and GEE, hence producing more robust estimates. However, our study had some limitations. First, the study excluded children aged below 15 years, the pregnant women and key populations. This means that the findings presented in this paper cannot be generalized to these sub-populations. Second, the analysis for the secondary outcome, mortality, did not account for the competing risk of loss to follow up, which could result in underestimation. An interrupted time series analysis where mortality and LTFU are considered as competing risks would help to improve our results on mortality. Third, we could not analyse the influence of CD4 count on retention and mortality because during UTT period, CD4 count was not obtained from many patients since this was no longer a requirement to initiate ART.

## Conclusions

In summary, our study has shown that, overall, UTT had an effect on retention and mortality among PLHIV enrolled and receiving HIV care from 11 HFs managed by TASO in Uganda. There was a marked reduction in retention over time after UTT was introduced with little to moderate reductions in mortality. Retention differed between some health facilities and it was highest in Rukungiri and lowest in Soroti. Retention was slightly higher in males than females as well as among younger people compared to the older ones. Mortality was higher among males and patients aged 50 years and above. We recommend that additional research be undertaken to understand factors facilitating or hindering success of UTT. The declining trend in retention reported in this paper has negative policy implications especially if we must attain the UNAIDS 2030 target of 95-95-95. We therefore recommend innovative interventions to check the declining trend in retention following the test and treat policy.

## Supporting information

S1 Data (ZIP)
